# Supplementary material for: Identification of MicroRNAs as Viable Aggressiveness Biomarkers for Prostate Cancer
Source: Biomedicines. 2021 Jun 5;9(6):646. doi: 10.3390/biomedicines9060646 (PMC8227559; doi:10.3390/biomedicines9060646)
Supplement: Supplementary file 1 [file biomedicines-09-00646-s001.zip › biomedicines-1232765-supplementary.pdf]

Martinez-Gonzalez, LJ, Sanchez-Conde, V, Gonzalez-Cabezuelo, JM. et al. Identification of miRNAs as viable aggressiveness biomarkers for prostate cancer.

Supplementary Figure S1. Selection of candidate miRNA normalizer assay.

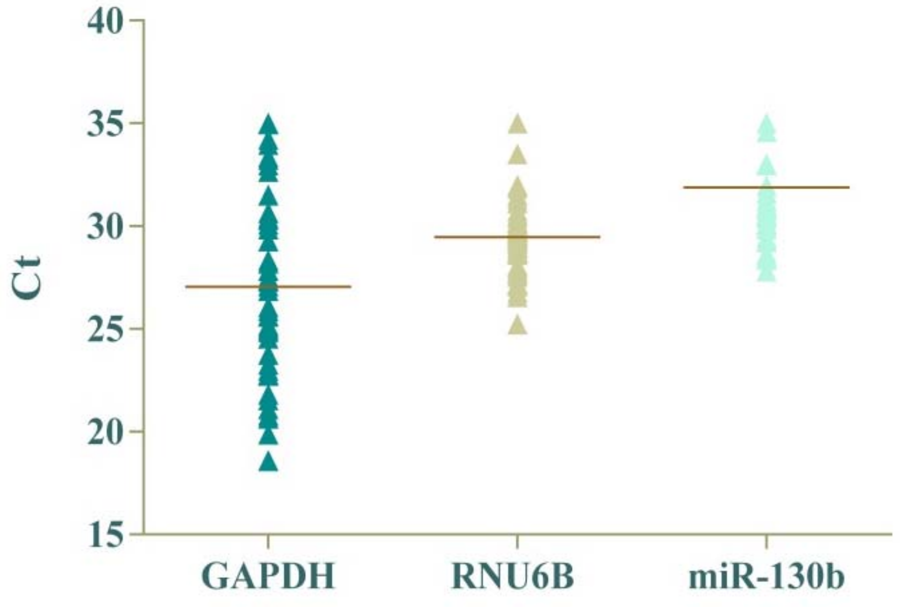

Footnote Figure S1. Cycle threshold (Ct) values of different tested endogenous controls (RNU6, GAPDH, and miR-130b) in tissue samples. After studying different normalization methods using these previous selected endogenous controls and all the ten miRNAs of present work, we concluded that normalization using RNU6B as control is the best option. In the graph it can be seen that RNU6B normalizer shows less variability and earlier Ct values than miR-130b

Supplementary Figure S2. Volcano Plot comparing non-tumoral samples (G0) with tumoral samples Gleason score ≤ 7 (G1).

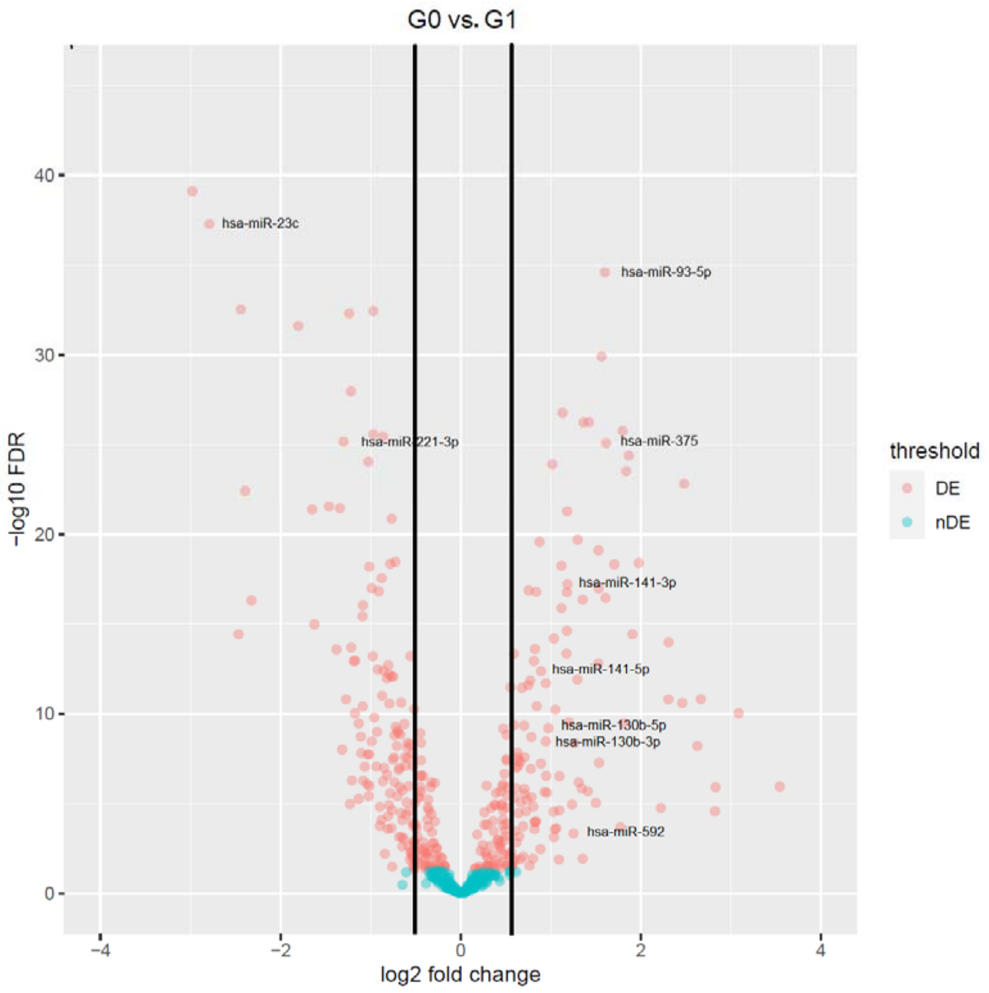

Footnote Figure S2. Volcano plot represents miRNAs dispersion according to their expression patterns; using logFC (X axis) versus statistical significance FDR (Y axis). In blue, they are represented those miRNAs that are not differentially expressed contrasting with DE analysis; which are represented in red. As nearer to the left side, miRNAs are under-expressed contrasting with those closer to the right side; which are over-expressed. Moreover, those miRNAs which are on the upper part of the Y axis have a stronger statistical significance. Most interesting miRNAs for present work are identified surrounded by a circle.

Supplementary Figure S3. Volcano Plot comparing non-tumoral samples (G0) with tumoral samples Gleason score > 7 (G2).

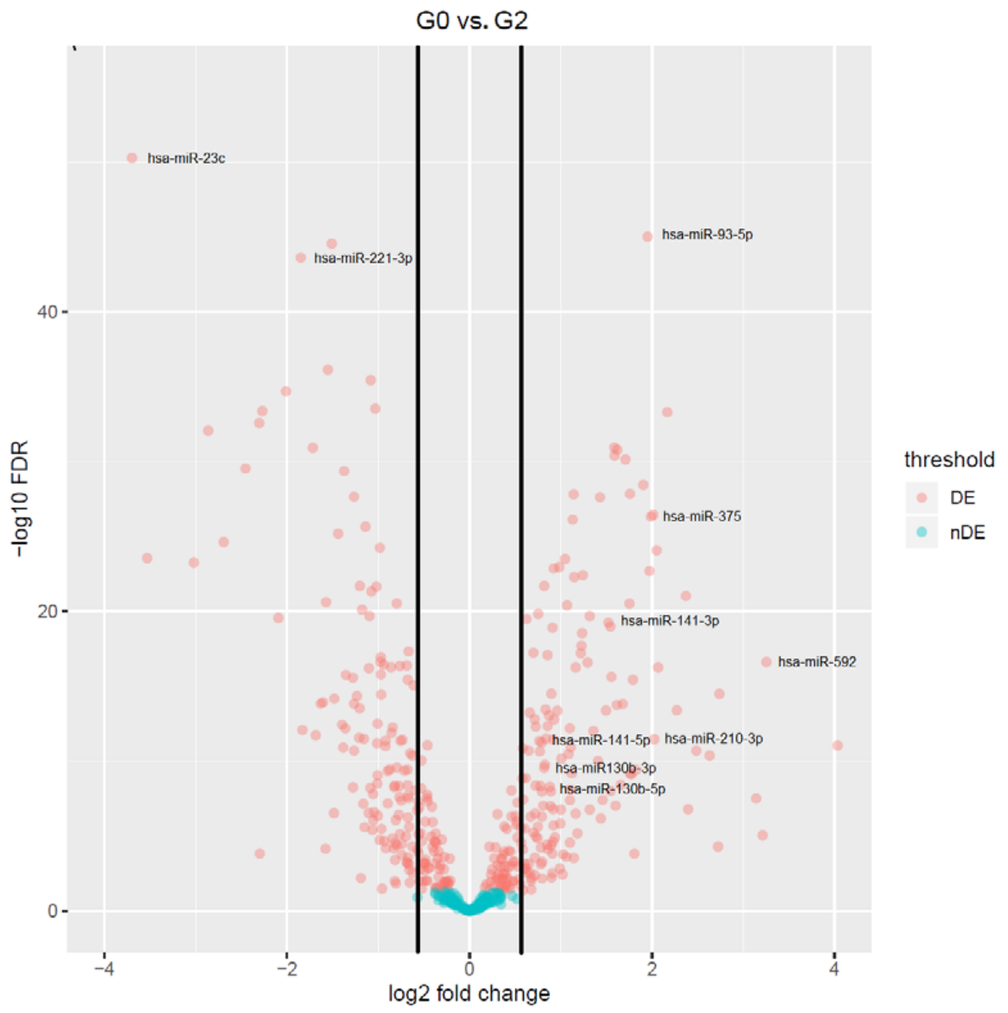

Footnote Figure S3. Volcano plot represents miRNAs dispersion according to their expression patterns; using logFC (X axis) versus statistical significance FDR (Y axis). In blue, they are represented those miRNAs that are not differentially expressed contrasting with DE analysis; which are represented in red. As nearer to the left side, miRNAs are under-expressed contrasting with those closer to the right side; which are over-expressed. Moreover, those miRNAs which are on the upper part of the Y axis have a stronger statistical significance. Most interesting miRNAs for present work are identified surrounded by a circle.

Martinez-Gonzalez, LJ, Sanchez-Conde, V, Gonzalez-Cabezuelo, JM. et al. Identification of miRNAs as viable aggressiveness biomarkers for prostate cancer.

Supplementary Figure S4. Expression patterns of miRNAs (141, 375 and 130b). a) Representing Gleason scores ≤7 versus Gleason scores >7; b) Representing T-Stage T1-T2 versus T3-T4.

a)

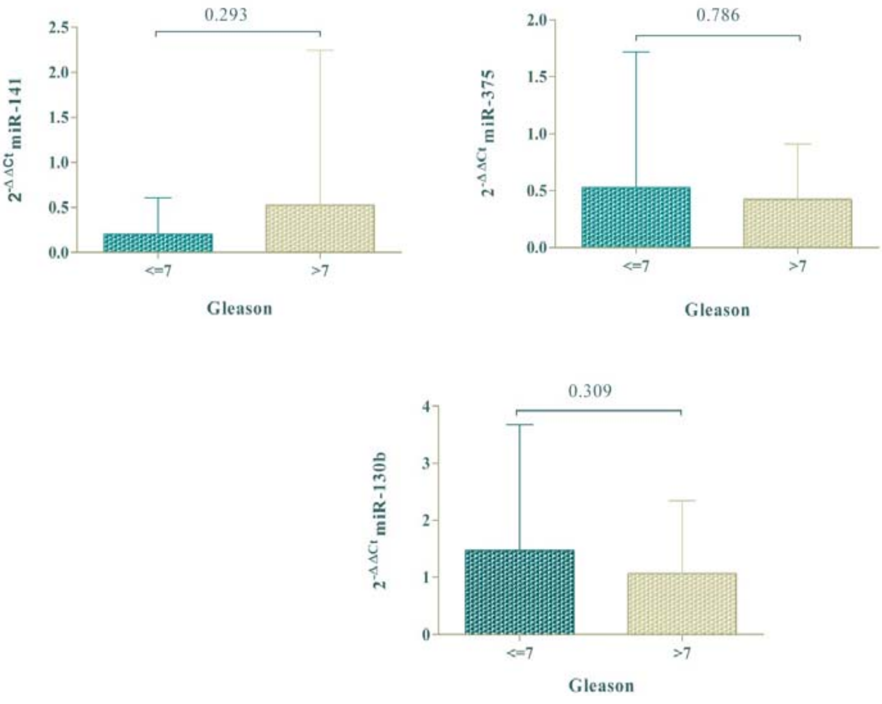

b)

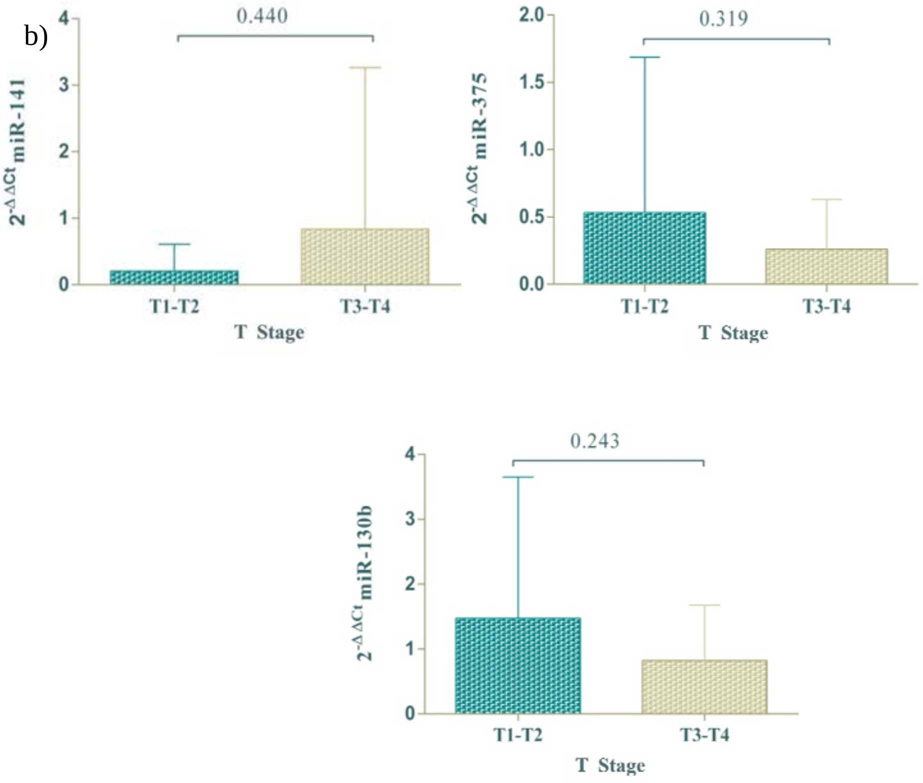

Supplementary Figure S5. Expression patterns of miRNAs (141, 375 and 130b) according to PSA values ranged in > 4 < 10 ng/ml, >10 < 20 ng/ml and > 20 ng/ml.

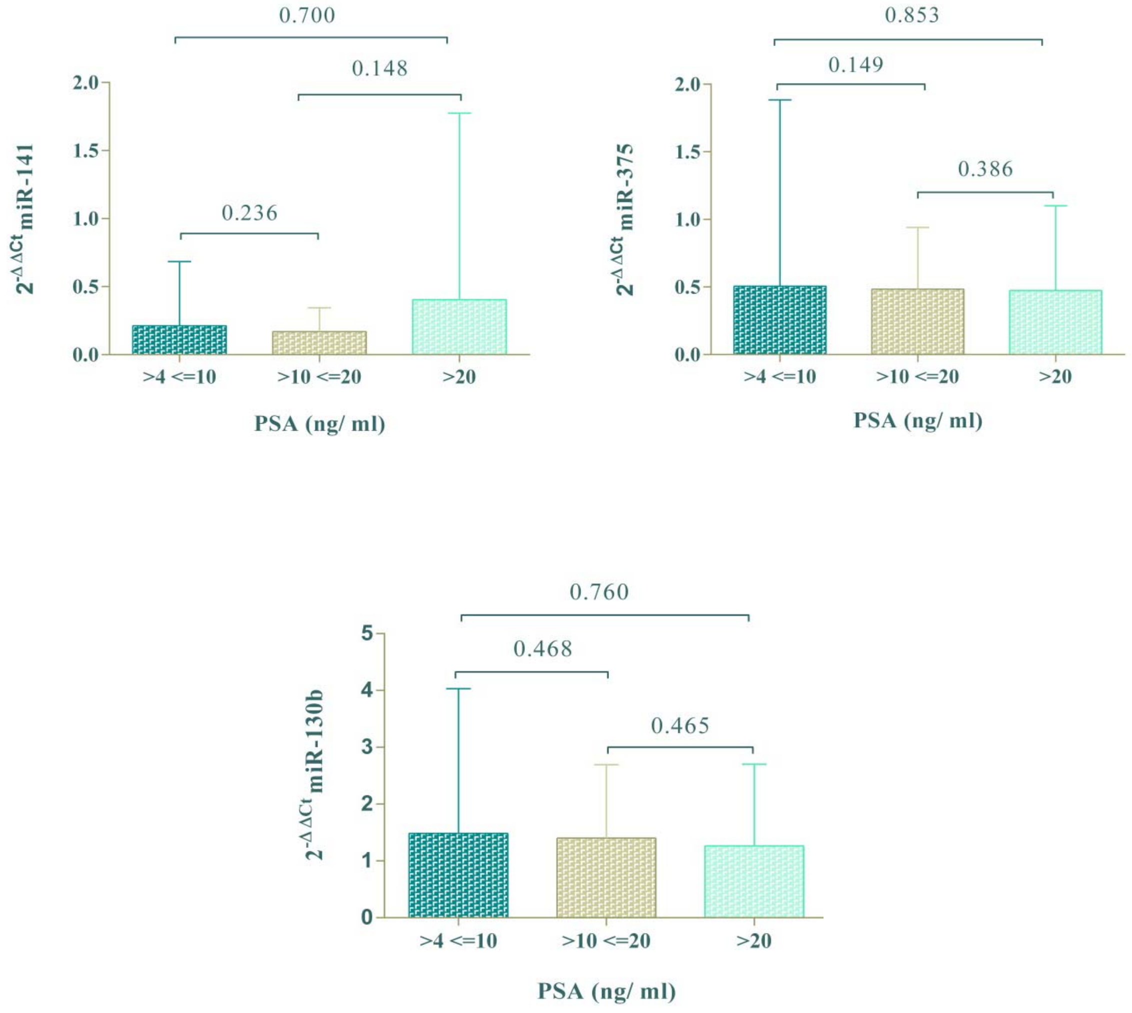

Supplementary Figure S6. miRNA 93-5p expression patterns in tissue and plasma using ΔCT normalisation with RNU6B.

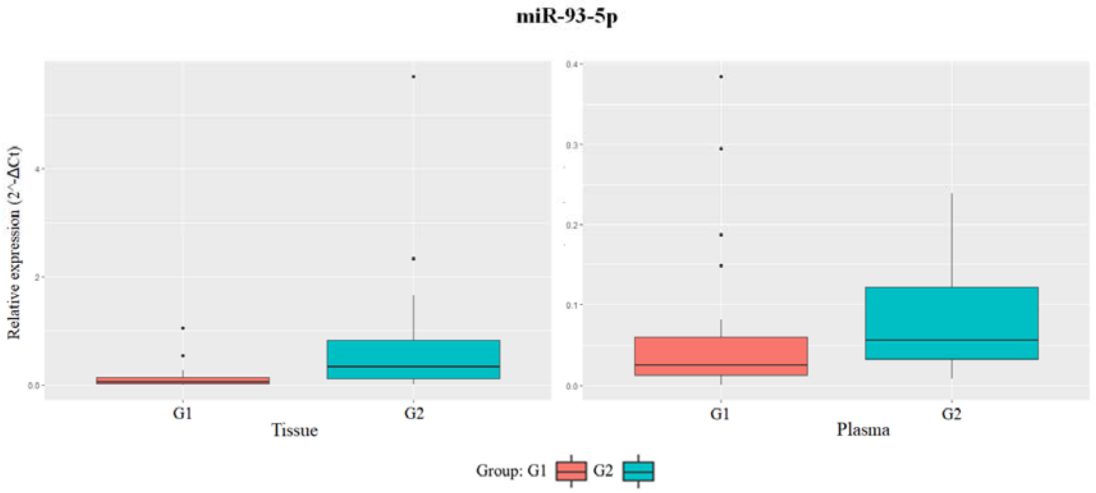

Footnote Figure S6. At the left we could see a significant over- expression pattern in miRNA 93-5p (<0.0001). At the right side, we could see as in plasma there is also an over- expression pattern, but with not significant values due to the sample size (n=60).

Martinez-Gonzalez, LJ, Sanchez-Conde, V, Gonzalez-Cabezuelo, JM. et al. Identification of miRNAs as viable aggressiveness biomarkers for prostate cancer.

Supplementary Figure S7. JAK-STAT signalling pathway.

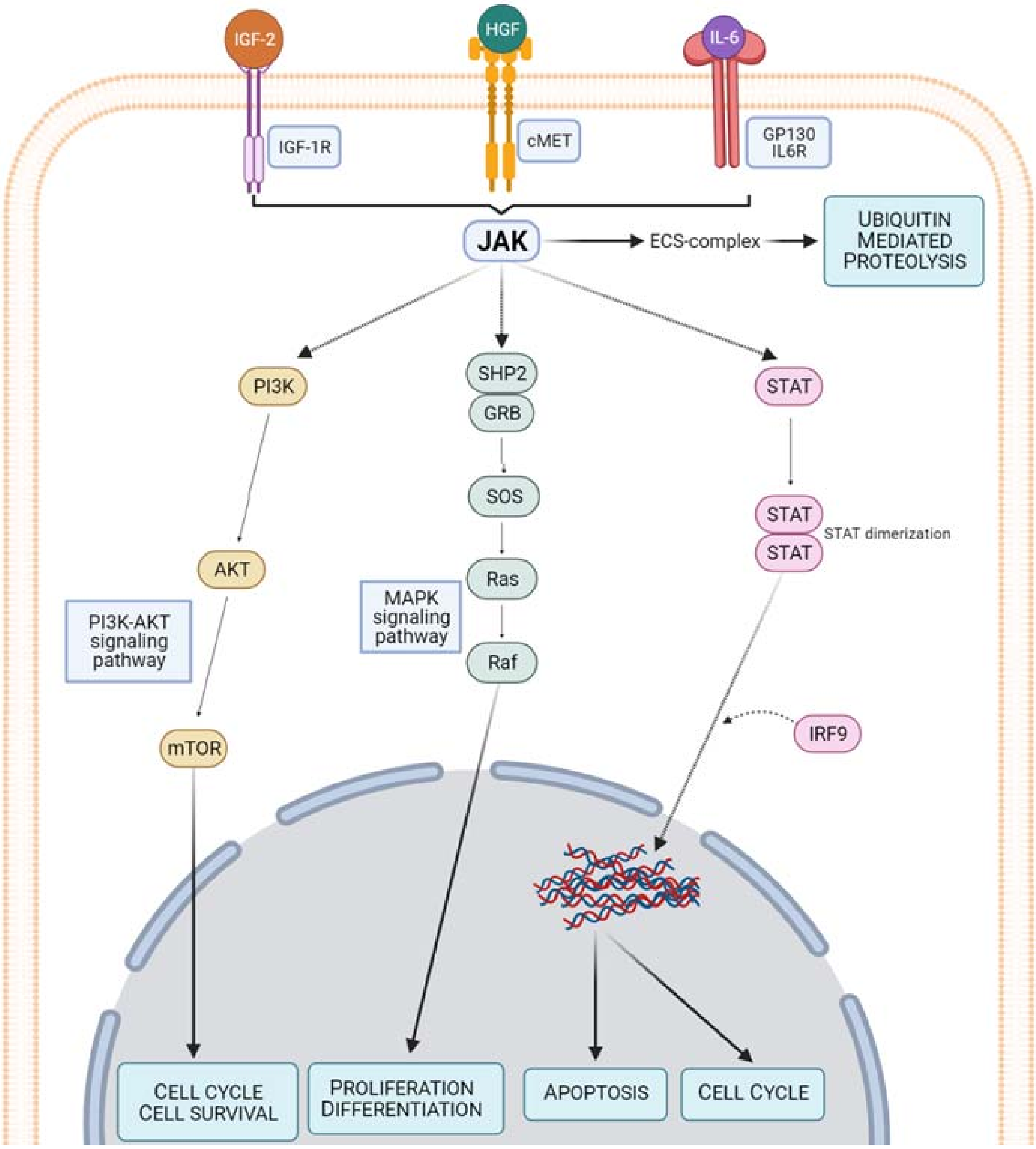

Supplementary Table S1. Values obtained for selection of the best housekeeping in tissue samples.

Gene	Ct	SD	CV
**GAPDH**	27.057	4.211	0.156
**RNU6B**	29.466	1.751	0.059
**miR-130b**	31.876	2.597	0.081

Footnote Table S1. SD (Standard Variation); CV (Variation coefficient); Ct (Cycle threshold).

Supplementary Table S2. Reported data of housekeepings according NormFinder and BestKeeper tools.

		NormFinder		***BestKeeper***	
**Gene**	Stability value	Standard Error	Range	Deviation	Range
**RNU6B**	0.734	0.431	1	1.15	1
**miR-130b**	1.418	0.263	2	1.22	2
**GAPDH**	2.670	0.286	3	3.29	3

Supplementary Table S3. Details of the probes.

Assay Name	Assay ID	Mature miRNA Sequence
**miR-592**	479075 mir	UUGUGUCAAUAUGCGAUGAUGU
**miR-23c**	478783 mir	AUCACAUUGCCAGUGAUUACCC
**miR-93-5p**	478210 mir	CAAAGUGCUGUUCGUGCAGGUAG
**miR-210-3p**	477970 mir	CUGUGCGUGUGACAGCGGCUGA
**miR-221-3p**	477981 mir	AGCUACAUUGUCUGCUGGGUUUC
**miR-141-5p**	478712 mir	CAUCUUCCAGUACAGUGUUGGA
**miR-375-3p**	478074 mir	UUUGUUCGUUCGGCUCGCGUGA
**miR-130b-3p**	477840 mir	CAGUGCAAUGAUGAAAGGGCAU

Supplementary Table S4. Candidate miRNAs for diagnostic biomarkers of PC (G0 vs G1)

miRNA	logFC	F	PValue	FDR
miR-891a-5p	−4.68071	524.75245	2.11794×10^{-81}	1.12463×10^{-78}
miR-1251-5p	−2.98162	222.13762	2.95098×10^{-42}	7.83485×10^{-40}
**miR-23c**	**−2.79229**	**209.27812**	**2.93773×10^{-40}**	**5.19979×10^{-38}**
**miR-93-5p**	**1.60176**	**191.57714**	**1.89048×10^{-37}**	**2.50961×10^{-35}**
miR-323b-3p	−2.44272	178.19999	2.79127×10^{-35}	2.96433×10^{-33}
miR-27b-3p	−0.97078	177.21132	4.05297×10^{-35}	3.58688×10^{-33}
miR-652-3p	−1.23890	175.95381	6.51806×10^{-35}	4.94442×10^{-33}
miR-204-5p	−1.80311	171.46500	3.5789×10^{-34}	2.37549×10^{-32}
miR-200c-3p	1.56315	160.82584	2.11939×10^{-32}	1.25044×10^{-30}
miR-889-3p	−1.21975	149.14740	2.0145×10^{-30}	1.06973×10^{-28}
miR-92a-3p	1.13074	141.95792	3.46116×10^{-29}	1.67083×10^{-27}
miR-17-5p	1.42355	138.77909	1.22922×10^{-28}	5.43928×10^{-27}
miR-148a-3p	1.36335	138.46089	1.39596×10^{-28}	5.70194×10^{-27}
miR-96-5p	1.79812	135.53316	4.51269×10^{-28}	1.71162×10^{-26}
miR-99b-3p	−0.97138	134.34153	7.28612×10^{-28}	2.57929×10^{-26}
miR-23b-3p	−0.86368	133.46958	1.03511×10^{-27}	3.43525×10^{-26}
**miR-221-3p**	**−1.30280**	**131.63828**	**2.16729×10^{-27}**	**6.76958×10^{-26}**
miR-20a-5p	1.61505	131.04489	2.75488×10^{-27}	8.12691×10^{-26}
**miR-375**	**1.86559**	**126.97730**	**1.43515×10^{-26}**	**4.01086×10^{-25}**
miR-145-3p	−1.02683	124.98426	3.23434×10^{-26}	8.58716×10^{-25}
**miR-141-3p**	**1.35452**	**81.02212**	**3.91529×10^{-18}**	**4.33129×10^{-17}**
**miR-141-5p**	**0.81289**	**62.63751**	**1.44426×10^{-14}**	**1.14463×10^{-13}**
**miR-130b-5p**	**0.97376**	**43.12139**	**1.22171×10^{-10}**	**6.17839×10^{-10}**
**miR-130b-3p**	**0.78417**	**40.54807**	**4.13977×10^{-10}**	**1.91149×10^{-09}**
**miR-592**	**1.25212**	**13.84071**	**0.000220054**	**0.000444291**

Footnote Table S4. Abbreviations: DE (Differential expression); logFC (logaritmic fold change); F (Quasi-likelihood F-statistic for the GLM (Quasi Likelihood F-test)); FDR (False Discovery Rate). Here comparisons were performed including tissue samples comparing non-tumoral area (G0; n = 51) with tumoral tissue area above Gleason ≤7 (G1; n = 285).

Supplementary Table S5. Candidate miRNAs for diagnostic biomarkers of PC (G0 vs G2).

miRNA	logFC	F	PValue	FDR
miR-891a-5p	−5.36828	525.99579	1.54729 x 10^{-81}	8.21611 x 10^{-79}
**miR-23c**	**−3.69722**	**298.41920**	**1.91778 x 10^{-53}**	**5.09171 x 10^{-51}**
**miR-93-5p**	**1.95193**	**260.37810**	**5.31929 x 10^{-48}**	**9.41515 x 10^{-46}**
miR-145-3p	−1.50865	256.42740	2.02380 x 10^{-47}	2.68660 x 10^{-45}
**miR-221-3p**	**−1.84835**	**249.41856**	**2.20268 x 10^{-46}**	**2.33925 x 10^{-44}**
miR-222-3p	−1.55122	200.09936	8.23706 x 10^{-39}	7.28980 x 10^{-37}
miR-23b-3p	−1.08182	195.40754	4.60195 x 10^{-38}	3.49091 x 10^{-36}
miR-204-5p	−2.01057	190.37724	2.94762 x 10^{-37}	1.95648 x 10^{-35}
miR-27b-3p	−1.03189	182.91807	4.74251 x 10^{-36}	2.79808 x 10^{-34}
miR-378d	−2.26930	181.64211	7.65033 x 10^{-36}	4.06232 x 10^{-34}
miR-96-5p	2.166465	180.90695	1.00810 x 10^{-35}	4.86638 x 10^{-34}
miR-133b	−2.30342	176.18918	5.96302 x 10^{-35}	2.63863 x 10^{-33}
miR-1251-5p	−2.86172	172.92863	2.05142 x 10^{-34}	8.37927 x 10^{-33}
miR-708-3p	1.587019	165.89458	3.00833 x 10^{-33}	1.14101 x 10^{-31}
miR-136-3p	−1.71603	165.60550	3.36134 x 10^{-33}	1.18991 x 10^{-31}
miR-200c-3p	1.618519	164.66236	4.82911 x 10^{-33}	1.60266 x 10^{-31}
miR-17-5p	1.588999	162.21678	1.23853 x 10^{-32}	3.86859 x 10^{-31}
miR-425-5p	1.708293	160.52788	2.37820 x 10^{-32}	7.01570 x 10^{-31}
**miR-375**	**1.988737**	**136.97024**	**2.53528 x 10^{-28}**	**4.98606 x 10^{-27}**
**miR-141-3p**	**1.522050**	**95.83539**	**6.34610 x 10^{-21}**	**5.91189 x 10^{-20}**
**miR-592**	**3.252722**	**81.48417**	**3.19623 x 10^{-18}**	**2.49588 x 10^{-17}**
**miR-210-3p**	**2.024351**	**53.96053**	**7.70099 x 10^{-13}**	**3.49506 x 10^{-12}**
**miR-141-5p**	**0.766069**	**53.28103**	**1.05448 x 10^{-12}**	**4.58960 x 10^{-12}**
**miR-130b-3p**	**0.777535**	**37.92062**	**1.44995 x 10^{-09}**	**4.78216 x 10^{-09}**
**miR-130b-5p**	**0.793059**	**27.76311**	**1.99271 x 10^{-07}**	**5.03871 x 10^{-07}**

Footnote Table S5. Abbreviations: DE (Differential expression); logFC (logaritmic fold change); F (Quasi-likelihood F-statistic for the GLM (Quasi Likelihood F-test)); FDR (False Discovery Rate). Here comparisons were performed comparing non-tumoral area (G0; n = 51) with tumoral tissue area above Gleason >7 (G2; n = 195).

Supplementary Table S6. Comparisons values between TCGA and qPCR analysis developed in biopsy and plasmas from peripheral blood.

	**TCGA**		**PVal**	**Biopsy**		**PVal**	**Plasmas**		**PVal**
**miR-210-3p**	G0vsG1	*Over-expressed (G1)*	**-**	G0vsG1	*Over-expressed (G1)*	**<0.0001**	G1vsG2	*Under-expressed (G2)*	-
	G0vsG2	*Over-expressed (G2)*	**7.700 x 10^{-13}**	G0vsG2	*Over-expressed (G2)*	**1.852 x 10^{-10}**			
	G1vsG2	*Over-expressed (G2)*	**1.894 x 10^{-23}**	G1vsG2	*Over-expressed (G2)*	**0.001**			
**miR-221-3p**	G0vsG1	*Under-expressed (G1)*	**2.167 x 10^{-27}**	G0vsG1	*Over-expressed (G1)*	**<0.0001**	G1vsG2	*Over-expressed (G2)*	-
	G0vsG2	*Under-expressed (G2)*	**2.202 x 10^{-46}**	G0vsG2	*Over-expressed (G2)*	**<0.0001**			
	G1vsG2	*Under-expressed (G2)*	**-**	G1vsG2	*Over-expressed (G2)*	**-**			
**miR-23c**	G0vsG1	*Under-expressed (G1)*	**2.937 x 10^{-40}**	G0vsG1	*Over-expressed (G1)*	**1.065 x 10^{-05}**	G1vsG2	*Over-expressed (G2)*	0.04654
	G0vsG2	*Under-expressed (G2)*	**1.917 x 10^{-53}**	G0vsG2	*Over-expressed (G2)*	**4.297 x 10^{-07}**			
	G1vsG2	*Under-expressed (G2)*	**1.609 x 10^{-07}**	G1vsG2	*Under-expressed (G1)*	**-**			
**miR-93-5p**	G0vsG1	*Over-expressed (G1)*	**1.890 x 10^{-37}**	G0vsG1	*Over-expressed (G1)*	**3.434 x 10^{-05}**	G1vsG2	*Over-expressed (G2)*	0.0594*
	G0vsG2	*Over-expressed (G2)*	**5.319 x 10^{-48}**	G0vsG2	*Over-expressed (G2)*	**2.123 x 10^{-11}**			
	G1vsG2	*Over-expressed (G2)*	**-**	G1vsG2	*Over-expressed (G2)*	**<0.0001**			
**miR-592**	G0vsG1	*Over-expressed (G2)*	**<0.0001**	G0vsG1	*Over-expressed (G1)*	**-**	G1vsG2	***ND***	**-**
	G0vsG2	*Over-expressed (G2)*	**3.196 x 10^{-18}**	G0vsG2	*Over-expressed (G2)*	**-**			
	G1vsG2	*Over-expressed (G2)*	**1.141 x 10^{-30}**	G1vsG2	*Over-expressed (G2)*	**-**			
**miR-130b-3p**	G0vsG1	*Over-expressed (G1)*	**4.139 x 10^{-10}**	G0vsG1	***NA***	**-**	*EA*		
	G0vsG2	*Over-expressed (G2)*	**1.449 x 10^{-09}**						
	G1vsG2	*Over-expressed (G2)*	**-**	G0vsG2	***NA***	**-**			
**miR-130b-5p**	G0vsG1	*Over-expressed (G1)*	**1.221 x 10^{-10}**						
	G0vsG2	*Over-expressed (G2)*	**1.992 x 10^{-07}**	G1vsG2	*Over-expressed (G2)*	**-**			
	G1vsG2	*Under-expressed (G2)*	**-**						
**miR-141-3p**	G0vsG1	*Over-expressed (G1)*	**3.915 x 10^{-18}**	G0vsG1	***NA***	**-**			
	G0vsG2	*Over-expressed (G2)*	**6.346 x 10^{-21}**						
	G1vsG2	*Over-expressed (G2)*	**-**	G0vsG2	***NA***	**-**			
**miR-141-5p**	G0vsG1	*Over-expressed (G1)*	**1.444 x 10^{-14}**						
	G0vsG2	*Over-expressed (G2)*	**1.054 x 10^{-12}**	G1vsG2	*Over-expressed (G2)*	**-**			
	G1vsG2	*Under-expressed (G1)*	**-**						
**miR-375**	G0vsG1	*Over-expressed (G1)*	**1.435 x 10^{-26}**	G0vsG1	*Over-expressed (G1)*	**-**			
	G0vsG2	*Over-expressed (G2)*	**2.535 x 10^{-28}**	G0vsG2	*Over-expressed (G2)*	**-**			
	G1vsG2	*Over-expressed (G2)*	**-**	G1vsG2	*Over-expressed (G2)*	**-**			

Foonote Table S6. PVal : T-test pvalue, NA: not analysed. ND: no data available. EA: Excluded from analysis after observing TCGA & Tissues results. Just those data with representative values are included (**-**), *except for the p-value of miR-93-5p analysis from plasmas from peripheral blood.
